# Morphological Diversification of the “Dasyphyllous” *Stipa* Species (Poaceae) from the Balkan Peninsula, with a Description of a New Species, *S. stevanoviciorum*

**DOI:** 10.3390/plants14071035

**Published:** 2025-03-27

**Authors:** Eva Kabaš, Jelica Novaković, Predrag Lazarević, Snežana Vukojičić, Vera Stanković, Dmitar Lakušić

**Affiliations:** 1 Institute of Botany and Botanical Garden “Jevremovac”, Faculty of Biology, University of Belgrade, 11000 Belgrade, Serbia; jelica@bio.bg.ac.rs (J.N.); predrag.lazarevic@bio.bg.ac.rs (P.L.); sneza@bio.bg.ac.rs (S.V.); dlakusic@bio.bg.ac.rs (D.L.); 2 Institute of Criminological and Sociological Research, 11000 Belgrade, Serbia; vera.batanjski@gmail.com

**Keywords:** *Stipa stevanoviciorum*, feather grasses, morphology, numerical analyses, Balkan Peninsula, Serbia, taxonomy

## Abstract

The interspecific relations that have been previously observed within the *Stipa dasyphylla* group are intricate and require further clarification. The aim of this study was to determine whether the specimens from Serbia deserve a separate taxonomic status. Various “dasyphyllous” *Stipa* species (those with hairy adaxial leaves) from the Balkan Peninsula were collected and analysed using morphological methods and microscopic observations of the macro- and micro-ornamentation of their leaves and lemmas. Based on analyses of 160 individuals from 17 populations belonging to three “dasyphyllous” *Stipa* species (*S. pontica*, *S. ucrainica*, and *S. dasyphylla*) which were collected in the central and eastern Balkan Peninsula (Serbia, North Macedonia, Bulgaria, Greece) and in Central Europe (Czech Republic, Slovakia, Hungary, Romania), *Stipa stevanoviciorum* is described as a new species from Serbia. This taxon includes the subspecies *stevanoviciorum*, which grows on ultramafic substrates, and the subspecies *pseudodasyphylla*, which is found on carbonate bedrock. These taxa differ from the closely related *S. dasyphylla* primarily in the length of their upper cauline leaves and the distance from the end of the dorsal line of the hairs to the top of the anthecium. The ecological and biogeographical characteristics of the taxa and a morphological comparison with similar species are given together with the key to the species’ identification. Images of key morphological characters are included, along with information on their distribution, habitat, and conservation implications.

## 1. Introduction

*Stipa* L. is one of the largest genera in the family Poaceae and the subfamily Pooideae [1]. Due to its very complicated taxonomy and unclear delimitation, as well as its uncertain number of species, the genus has always been considered obscure [2]. According to the current narrow approach, the genus encompasses approximately 150 species distributed throughout Asia, Europe, and North Africa, with the greatest diversity being found in the open grasslands and steppes of the warm temperate zones of the Old World [3,4,5,6,7,8,9,10,11,12]. To date, there are many unresolved taxonomic issues within the different taxonomic levels of this genera, yet new species continue to be described, mainly species from Asia but also species from Africa and Europe [11,12,13,14,15,16,17,18,19,20,21,22,23,24,25].

The nominal section of the genus *Stipa* L., which is widely regarded as one of the most taxonomically problematic genera, comprises up to 55 species that depend on various concepts [5,7,8,12,21,26,27,28,29,30,31,32,33]. Furthermore, one of its supposedly taxonomically sound and well-differentiated groups—the series *Dasyphyllae* Martinovský (= “dasyphyllous” *Stipa* species)—has turned out to be very intricate, encompassing Central- and Eastern-European species, as well as those distributed in the Caucasus, Siberia and Asia. The species of this series represent a natural, clearly defined group of feather grasses within the section *Stipa*, which is primarily characterized by its leaf ornamentation, i.e., the leaf surface of these species is never bare. The indumentum elements appear in different forms, such as hairs, bristles, conical papillae, or transitional structures in between these. These indumentum structures tend to vary, amongst the species of this group, in length, frequency, and the size of the angle they form with the leaf surface. They actually represent homologous and evolutionarily ancient structures of epidermal origin that have an important taxonomic value, and indicate the natural specificity of this group [33]. Despite all of these characteristics, the interspecific relations within this group are intricate and require further clarification (for details see [34]).

Based on the key and the descriptions given in *Flora Europea* [7], the *S. dasyphylla* group comprises four species: *S. dasyphylla* (Lindem.) Czern. ex Trautv., *S. zaleskii* Wilensky, *S. pontica* P. A. Smirn., and *S. ucrainica* P. A. Smirn, although there are different interpretations regarding the representatives of this group [30,34,35]. The nominal species of the series, *S. dasyphylla*, has been known in Europe for the longest time. It was the first representative of the complex to appear in the botanical literature [34]. The type specimen was collected in 1853 by Chernyaev in Ukraine, Kharkov, on knolls and hills of Rogan [32]. The ornamentation of the leaf blade (both on the vegetative and cauline leaves) is a constant feature that clearly distinguishes *S. dasyphylla* from other species of the group. *S. dasyphylla* has leaves whose abaxial surface is covered with abundant long soft hairs over their entire length, while the adaxial surface also bares sparse or abundant long, more or less soft hairs throughout its whole length [32]. The presence of these soft hairs over the whole leaf length makes *S. dasyphylla* one of the most easily recognizable species within the S. *dasyphylla* group. The other species of the *S. dasyphylla* group, *S. ucrainica* and *S. zalesskii* (including *S. pontica*), are known to grow in the Balkan Peninsula [7,36,37]. On the other hand, the distribution of *S. dasyphylla* in the Balkans was not previously clear. This was mainly due to the fact that Martinovský [34], in his earlier work, considered *S. dasyphylla* as an aggregate species comprising three taxonomically closely related microspecies that occur in the relic central- and southeastern- European steppes, and considered that misidentifications were likely to happen. This species is generally distributed from Central Asia (north to southern part of western Siberia), across the European part of Russia, to Central Europe (west to Switzerland and Germany) [32,38]. In the Balkan Peninsula, it has so far been recorded at one site in Greece [32] and most recently in Serbia [39]. Valdés et al. [38] have also reported its observation in Bulgaria, but on the basis of personal communication with H. Scholz from 2003.

In this study, we focused on populations of *S. dasyphylla* from Serbia and other geographically close populations of “dasyphyllous” *Stipa* species in the central and eastern Balkan Peninsula and Central Europe. We performed classical morphometric analyses to gain insight into the diversity of the populations studied. The main aim of this study was to determine whether the populations of *S. dasyphylla* from Serbia are morphologically outstanding or not.

## 2. Results

In our research on the distribution and diversity of the genus *Stipa* in the Balkan Peninsula, we have found only a few populations that belong to the “dasyphyllous” Stipa species. By comparing and examining plants during fieldwork, as well as the available herbarium specimens, we found that the most morphologically striking and diverse populations are plants from Serbia that were previously identified as *S. dasyphylla*.

### 2.1. Morphometric Analyses

**Figure 1:**
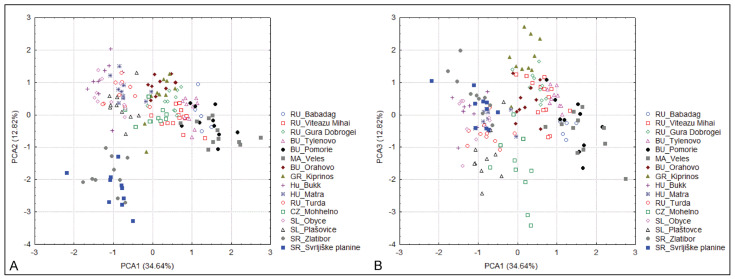
Principal component analysis (PCA) of 160 individuals from 17 populations belonging to three species of “dasyphyllous“ Stipa from the Balkan Peninsula based on 20 morphometric characters. (**A**) the two first PCA axes, (**B**) the first and third PCA axes. The acronyms in the figure represent the country code and site toponym: BU—Bulgaria, GR—Greece, HU—Hungary, RU—Romania, CZ—Czech, SL—Slovenia, SR—Serbia.

The PCA shows that, with the exception of the populations from Serbia, the other populations show only a very low degree of separation. Along the first two principal component axes, the populations of *S. dasyphylla* from Serbia are completely separated, while the other populations of *S. dasyphylla*, from Central Europe, are associated with *S. ucrainica* and *S. pontica*, which largely overlap. Along the first and third discriminant principal component axes, populations of *S. dasyphylla* from Central Europe overlap with populations of *S. dasyphylla* from Serbia. At the same time, this project completely separates the populations of *S. dasyphylla* from the largely overlapping *S. ucrainica* and *S. pontica* populations (Figure 1). The characters that contribute most to the observed structure of variability are the plant height (PH), panicle length (LP), number of flowers in the panicle (NF), length of vegetative shoots (LV), basal leaf-blade diameter (BASD), length of the anthecium (AL), distance from the end of the dorsal line of the hairs to the top of the anthecium (DDL), and distance from the end of the ventral line of the hairs to the top of the anthecium (DVL).

**Figure 2:**
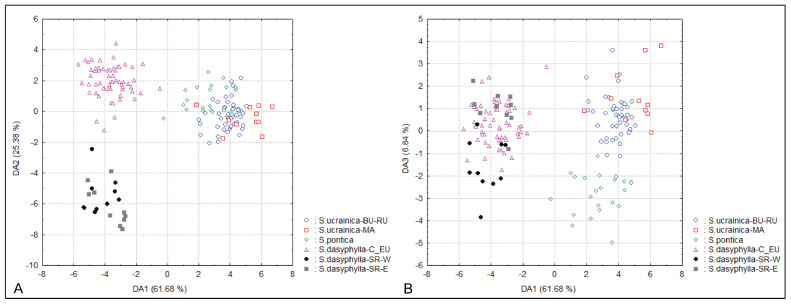
Canonical discriminant analyses of 160 individuals from 17 populations belonging to three species of “dasyphyllous” Stipa from the Balkan Peninsula, based on 20 morphometric characters. All individuals are included in six groups identified a priori as potential OTUs based on their taxonomic and biogeographic affiliations. (**A**) the two first discriminant axes, (**B**) the first and the third discriminant axes. The acronyms in the figure represent the combination of the species name and biogeografic affiliation of the group: BU—Bulgaria, C_EU—central Europe, MA—North Macedonia, RU—Romania, SR-E—eastern Serbia, SR-W—western Serbia.

The CDA that was performed on six groups of individuals identified a priori as potential OTUs based on their taxonomic and biogeographic affiliations not only confirmed the results of the PCA, i.e., the complete morphological segregation of *S. dasyphylla* from Serbia, but also the segregation between Central European *S. dasyphylla*, *S. ucrainica* and *S. pontica* along the first three discriminant axes (Figure 2). In addition, the CDA revealed a significant morphological diversification between the western and eastern Serbian populations of *S. dasyphylla*. On the third discriminant axis, the Illyrian populations from the ultramafic rocks of western Serbia (*S. dasyphylla*-SR-W) and the Moesian populations from the limestones of eastern Serbia (*S. dasyphylla*-SR-E) are almost completely separated from each other. At the same time, the CDA showed extensive overlap between Danubian populations of *S. ucrainica* from Bulgaria and Romania (S. ucrainica-BU-RU) and Macedonian populations of *S. ucrainica* from North Macedonia (S. ucrainica-MA) (Figure 2).

**Figure 3:**
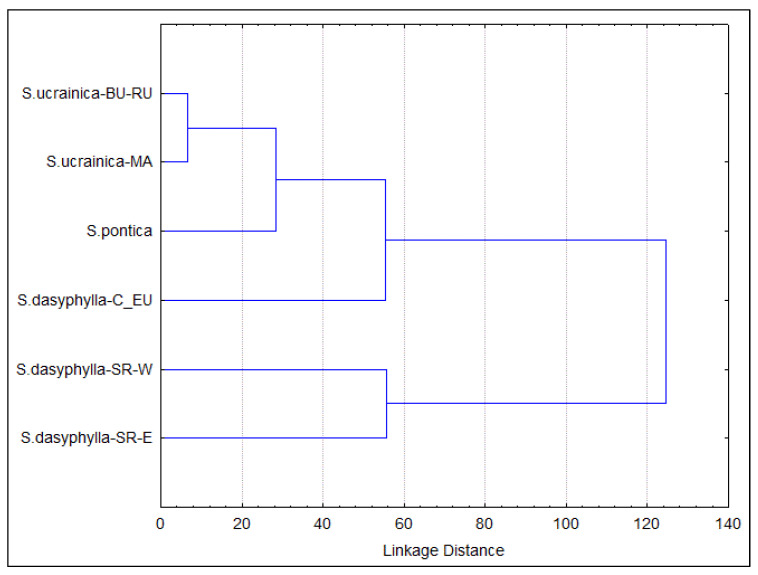
Overall similarities among morphological groups of “dasyphyllous” *Stipa* from the Balkan Peninsula based on the use of the agglomerative UPGMA clustering method on morphometric data. All individuals are included in six groups identified a priori as potential OTUs based on their taxonomic and biogeographic affiliations. The acronyms in the figure represent the combination of the species name and biogeografic affiliation of the group: BU—Bulgaria, C_EU—central Europe, MA—North Macedonia, RU—Romania, SR-E—eastern Serbia, SR-W—western Serbia.

The UPGMA cluster analysis revealed that the degree of morphological diversification between the eastern and western populations of *S. dasyphylla* in Serbia is extremely high. In contrast, the morphological differentiation between the Macedonian and Bulgarian-Romanian populations of *S. ucrainica* in the Black Sea hinterland is minimal. At the same time, *S. pontica* and the Central European *S. dasyphylla* show a moderate degree of segregation (Figure 3).

The classification function based on morphological characters revealed that, with the exception of the Macedonian (*S. ucrainica*-MA) and the Bulgarian-Romanian populations of *S. ucrainica* (*S. ucrainica*-BU-RU), all other individuals were assigned to their respective a priori-defined groups. The Macedonian individuals (*S. ucrainica*-MA) were correctly classified in 72.73% of cases, while 93.88% of the individuals of the Bulgarian-Romanian populations of *S. ucrainica* were correctly classified (Table 1).

**Figure 4:**
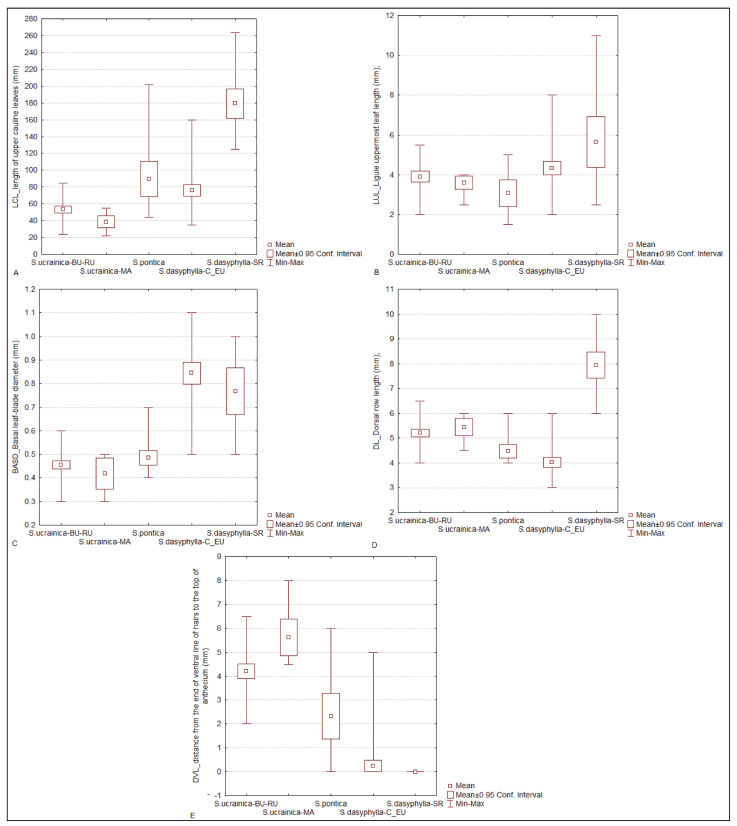
Box-and-whisker plots showing variation in four most significant factors contributing to the divergence among morphological groups of “dasyphyllous” *Stipa* from the Balkan Peninsula revealed obtained by discriminant function analysis (DFA). (**A**) length of upper cauline leaves (LCL), (**B**) ligule uppermost culm leaf length (LUL), (**C**) dorsal row length (DL), (**D**) basal leaf-blade diameter (BASD), (**E**) distance from the end of ventral line of hairs to the top of anthecium (DVL). The whiskers represent extreme values, the boxes include 0.95% of confidence interval, and small squares indicate the mean values.

The discriminant function analysis (DFA) showed that the most significant factors contributing to this divergence were the following characters: the length of the upper cauline leaves (LCL), ligule uppermost culm leaf length (LUL), dorsal row length (DL), distance from the end of the ventral line of the hairs to the top of the anthecium (DVL), and basal leaf-blade diameter (BASD) (Table 2, Figure 4).

### 2.2. Microscope Observations

**Figure 5:**
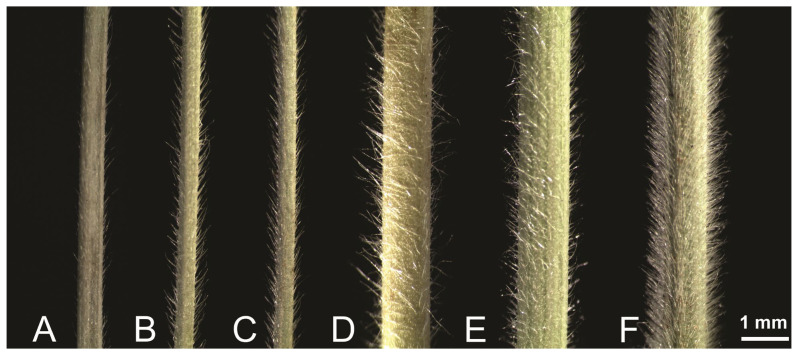
Abaxial surface of the vegetative leaf blades of: (**A**) *S. ucrainica*—Bulgaria, (**B**) *S. ucrainica*—North Macedonia, (**C**) *S. pontica*—Greece, (**D**) *S. dasyphylla*—Slovakia, (**E**) *S. stevanoviciorum* subsp. *pseudodasyphylla*—Serbia: Svrljiške, (**F**) *S. stevanoviciorum* subsp. *stevanoviciorum*—Serbia: Zlatibor.

The most striking qualitative differences that were observed relate to the ornamentation of the leaf indumentum, i.e., the quality and appearance of the hairs on the abaxial leaf surface, and the leaf diameter. Indeed, the collected specimens marked as *S. dasyphylla* have a larger leaf diameter and are equipped with long soft hairs that make them soft to the touch (Figure 5D–F). The densest, longest, and most abundant leaf hairs were observed in specimens from Serbia, especially those from Mount Zlatibor (Figure 5A), followed by specimens from Mount Svrljiške (Figure 5E).

In contrast, the specimens of *S. ucrainica* and *S. pontica* had thinner leaves with more or less appressed, shorter hairs that were less abundant and not soft to the touch (Figure 5A–C).

### 2.3. Taxonomic Treatment

Taking all of our results into account, we provide a detailed description and map the distribution of a new species, *S. stevanoviciorum*, with two subspecies: *S. stevanoviciorum* subsp. *stevanoviciorum* and *S. stevanoviciorum* subsp. *pseudodasyphylla* subsp. nov. In the morphological descriptions, the value ranges correspond to the mean ± standard deviation, with the minimum and maximum values given in brackets.

*Stipa stevanoviciorum* Kabaš & D. Lakušić, sp. *nova*

**Figure 6:**
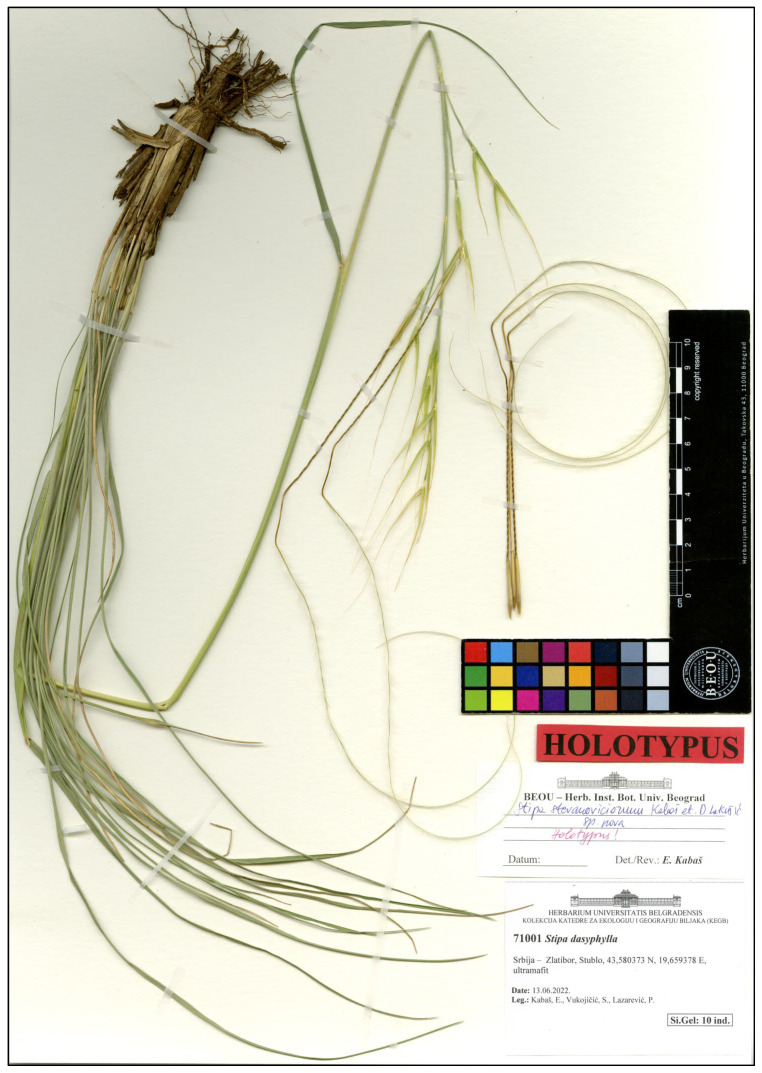
Holotype of *S. stevanoviciorum* subsp. *stevanoviciorum*.

Holotype: Serbia, Zlatibor Mt., Stublo, near Dubrava Monastery, 43.5806821 N, 19.6596977 E, 859 m, rocky grassland, serpentinite, 1 June 2021, Coll. E. Kabaš, P. Lazarević (BEOU 17763) (Figure 6).

Diagnosis: *Stipa stevanoviciorum* is most similar to *S. dasyphylla*, but differs in the following key characters: the culm ornamentation (pubescent vs. scabrous, rarely pubescent), the panicle basal internode surface (pubescent vs. scabrous, rarely pubescent), the upper sheaths ornamentation (glabrous to papilose vs. scabrous to pubescent), the basal leaf ligule margin (glabrous vs. ciliate to ciliolate, rarely glabrous), the length of the upper cauline leaves (125–264 mm vs. 35–160 mm), the length of the dorsal row of lemma hairs (6–10 mm vs. 3–6 mm); anthecium (22–25 mm vs. 19–22 mm), and several other morphological traits (Table 3).

**Figure 7:**
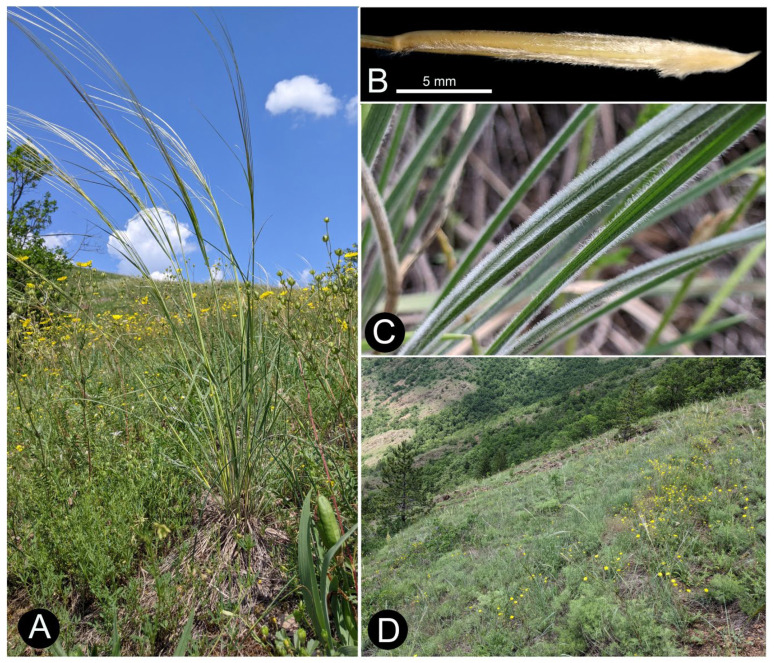
*Stipa stevanoviciorum* subsp. *stevanoviciorum* on classical site on Zlatibor Mt. (**A**) habitus, (**B**) anthecium with callus, (**C**) basal leaves, (**D**) habitat (photograph by Predrag Lazarević.).

Description: The plant is perennial, tufted, with a few culms and numerous vegetative shoots (Figure 7A,B), (22–)32–52(–59) cm tall, mostly four-noded, glabrous at nodes, and scabrous (the lower internodes) to more or less densely pubescent below them (the upper internodes). The leaves of the vegetative shoots: the sheaths of the external and internal leaves are mostly glabrous, the ligules are membranous and acute or slightly obtuse, the glabrous internal blades are convolute, the external blades are convolute to flat, and the leaves are green to pale green, are (39–)42–59(–70) cm long, are 1 mm in diameter, have an abaxial surface that is densely and softly hairy, and an adaxial surface pubescent or sparsely pilose. Cauline leaves: the sheaths are smooth to slightly papilose (usually in upper part of sheath), the ligules of the upper cauline leaves are (2.5-)2.9–8.3(-11) mm long, acute at the apex with very short cilia, and hairy on the back and margins, the blades are flat and are green or pale green, the uppermost culm is (12–)14–21(–27) cm long, and the abaxial surface is hairy and scabrous. The panicle is (7–)7–14(–18) cm long, exerted to partially enclosed, with (4–)5–10(–12) spikelets, branches with patent long and short hairs. The glumes are subequal and narrowly lanceolate, and the lower glume is (61–)66–92(–120) mm long while the upper glume is (54–)57–83(–110) mm long. The anthecium is (21–)22.1–24.3(–25.5) mm long, (1–)1.3–1.8(–2) wide, and callus with hairs, and the foot of the callus is curved, the peripheral ring is flattened (1–)1–1.3–1.5 × 0.5 mm, the lemma is straw-coloured, with seven lines of hairs, the dorsal and subdorsal lines are slightly fused at the base, the ventral line reaches the top of lemma, the dorsal line terminates (8–)9.3–11.6(–13) mm below the top of lemma length, the awn is (273–)295–364(–435) mm long, bigeniculate, the column is smooth and glabrous, twisted, brown or yellow, and (81–)88–101(–108) mm long, and the seta is (186–)204–265(–327) mm long and pilose, with (5–)5.3–6.8(–7.5) mm long hairs that gradually decrease in length towards the apex. 

Etymology: the new species is named in honour of our teachers Prof. dr Vladimir Stevanović and Prof. dr Branka Stevanović, Serbian botanists known for their contributions to the study of Balkan plants, especially the Pontic flora and the vegetation of the Pannonian and Central Balkan rocky steppes.

**Figure 8:**
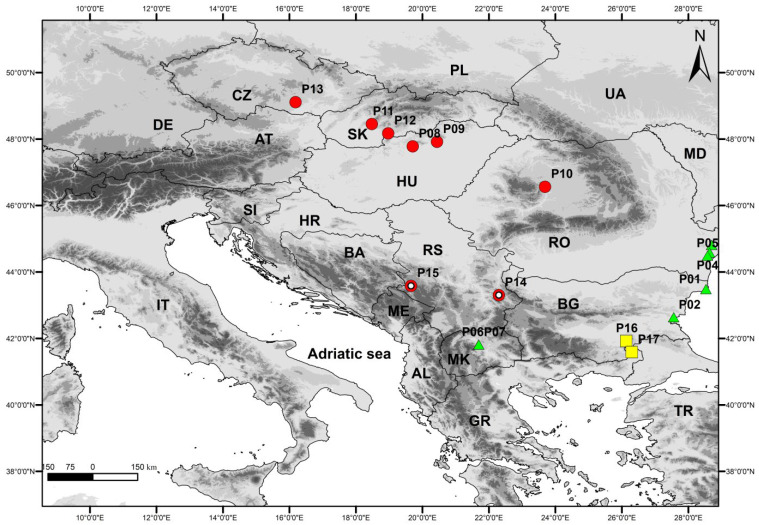
Studied populations of *Stipa ucrainica* (green triangle), *S. dasyphylla* (red circles), *S. stevanoviciorum* (red circles with white dots), and *S. pontica* (yellow sqares). Data on populations can be found in Table 1.

Distribution and habitat: *Stipa stevanoviciorum* is an endemic taxon, known so far only from Mount Zlatibor in western Serbia and from Mount Svrljiške in eastern Serbia (Figure 8). It grows on a xerophilous rocky grassland in the mountain rocky steppe vegetation of the class Festuco-Brometea Br.-Bl. et Tx. ex Soó 1947. The plants from Zlatibor Mt. in western Serbia inhabit the ultramafic substrate and contribute to the vegetation composition of the rocky steppe vegetation of the order Halacsyetalia sendtneri Ritter-Studnička 1974 (Figure 7D), while the plants from Svrljiške Mt. in eastern Serbia grow on a carbonate rocky grassland that occurrs in the mountain steppe vegetation of the order Festucetalia valesiacae Soó 1947.

Phenology: flowering and fruiting period: May–August.

Conservation status: At present, only about 100 flowering individuals have been recorded on Zlatibor Mt. in western Serbia, and approximately the same number of individuals have been recorded on Svrljiške Mt. in eastern Serbia, occupying a very small area of dry rocky grasslands. Due to its small population size and limited distribution, *S. stevanoviciorum* should be considered critically endangered (CR B2aci,ii,iii). The proximity of the road and the lack of grazing in that area, together with the closure of vegetation cover, may pose a threat to the survival of the very small populations of *S. stevanoviciorum*. The survival of these populations depends on the conservation of their habitats through national legislation, active in situ conservation (such as the preservation of open grasslandsand ex situ conservation measures, including seed banks and in vitro propagation.

#### Infraspecific Variability

*Stipa stevanoviciorum* subsp. *stevanoviciorum*

Description: The plant is perennial and tufted, with a few culms and numerous vegetative shoots (Figure 7), and is (35.3–)40.7–56.4(–59.5) cm tall, its blades are convolute to flat, silvery pale green, (417–)427–584(–698) cm long, and 1 mm in diameter, with an abaxial surface that is densely and softly hairy and an adaxial surface that is pubescent or sparsely pilose. Its panicle is (8.5–)9.2–16.9(–18.5) cm long and exerted, with (7–)7.2–10.8(–12) spikelets. Its glumes are subequal and narrowly lanceolate. Its anthecium is (21–)21.9–24.6(–25.5) mm long and callus with hairs, with a straw-coloured lemma that has seven lines of hairs, and its dorsal and subdorsal lines are slightly fused at the base, with the ventral line reaching the top of the lemma, the dorsal line terminating (9–)9.9–11.9(–13) mm below the top of lemma length, the awn being (273–)294.4–348.6(–357) mm long and bigeniculate, the column being smooth, glabrous, twisted, brown, and (81–)84.2–97.2(–102) mm long, and the seta being (186–)207–254(–268) mm long and pilose, with its hairs gradually decreasing in length towards the apex.

Distribution and habitat: *Stipa stevanoviciorum* subsp. *stevanoviciorum* is an endemic taxon, hitherto known only from Zlatibor Mt. in western Serbia and occurring on the ultramafic rocky grassland in the mountain rocky steppe vegetation of the order Halacsyetalia sendtneri at about 860 m. asl. (Figure 7C). The species occurs in a small area near the dirt road. The following species dominate the community at the classic site: *Stipa pulcherrima* C. Koch, *Festuca valesiaca* Schleicher ex Gaudin, *Danthonia alpina* Vest, *Artemisia alba* Turra, and *Asperula purpurea* (L.) Ehrend., while the following serpentinophyte species co-existing with them are the characteristic elements of the vegetation of the order Halacsyetalia sendtneri: *Euphorbia glabriflora* Vis., *Halacsya sendtneri* (Boiss.) Dörfl., *Iris reichenbachii* var. *bosniaca* Beck, *Paragymnopteris marantae* (L.) K.H. Shing, *Potentilla visianii* Pančić, *Silene armeria* L., *Silene paradoxa* L., *Stachys recta* subsp. *baldaccii* (K.Malý) Hayek, etc.

Phenology: flowering and fruiting period: May–August.

Conservation status: So far, just over 100 flowering individuals have been counted in a very small dry grassland area on Zlatibor Mt.; hence, the new subspecies should be considered as a critically endangered taxon (CR).

*Stipa stevanoviciorum* subsp. *pseudodasyphylla* Kabaš & D. Lakušić, subsp. *nova*

**Figure 9:**
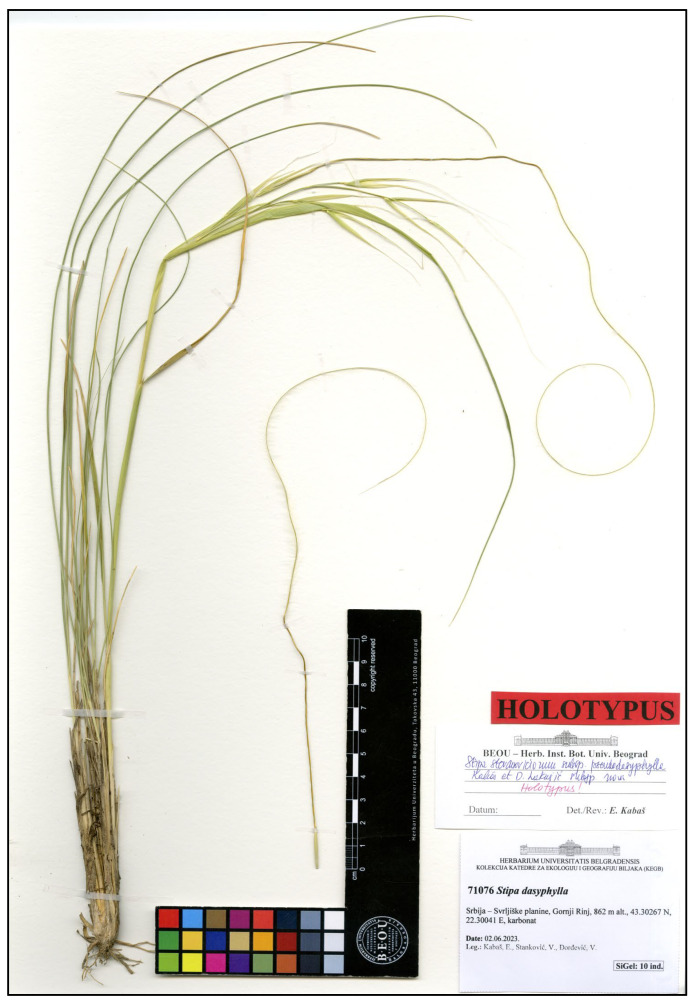
Holotype of *Stipa stevanoviciorum* subsp. *pseudodasyphylla*.

Holotype: Serbia, Svrljiške mountain, Gornji Rinj, 43.30267 N, 22.30041 E, 862 m, carbonate, 2 June 2023, Coll. E. Kabaš, V. Stanković, V. Đorđević (BEOU 71076) (Figure 9).

Diagnosis: Similar to *Stipa stevanoviciorum* subsp. *Stevanoviciorum*, from which the subspecies differs mostly in the following characters: plant height (223–460 mm vs. 353–595 mm); number of flowers in a panicle (4–12 vs. 7–12); length of the upper cauline leaves (125–190 mm vs. 159–264 mm), length of the ligule of the uppermost culm leaf (6–11 mm vs. 2.5–4 mm; upper sheath ornamentation—glabrous to papilose vs. papilose; basal ligule length (2–4 mm vs. 1–2 mm); basal ligule tip—(cilate vs. ciliolate); type of basal leaves in sward—in some individuals only flat or only convolute leaves are present vs. both flat and convolute leaves are always present; colour of the column—yellow vs. brown; and somewhat denser, longer, and softer hairs on the abaxial side of the leaf blades (Table 3).

Description: The plant is perennial and tufted, with a few culms and numerous vegetative shoots (Figure 9), is (22.3–)28–42.4(–46) cm tall, has blades that are convolute to flat, green, (394–)412–604(–699) mm long, and 1 mm in diameter, has an abaxial surface that is densely and softly hairy, and has an adaxial surface that is pubescent or sparsely pilose. The panicle is (6.6–)7–11.8(–15.5) cm long and exerted, with (4–)4–8.6(–12) spikelets. The glumes are subequal and narrowly lanceolate. The anthecium is (21.5–)22.3–24(–24) mm long and callus with hairs, with a lemma that is straw-coloured, has seven lines of hairs, has dorsal and subdorsal lines that are slightly fused at the base, has a ventral line reaching the top of the lemma, has a dorsal line terminating (8–)8.9–11.3(–12) mm below the top of the lemma length, has an awn that (287–)296.8–378.4(–435) mm long, has a bigeniculate column that is smooth, glabrous, twisted, yellow, and (93–)93.2–103.4(–108) mm long, and has seta that are (193–)203–276(–327) mm long and pilose, with the hairs gradually decreasing in length towards the apex.

Etymology: The name of the subspecies agrees with the name of the closely related and very similar *Stipa dasyphylla*, to which group it undoubtedly belongs. However, the similar habitus together with some significant differences in certain morphological features led us to add the prefix pseudo- to the species attribute *dasyphylla*, as we consider it appropriate for the name of the new taxon.

Distribution and habitat: *Stipa stevanoviciorum* subsp. *pseudodasyphylla* is an endemic taxon that is only known from Svrljiške Mt. in eastern Serbia. The subspecies occurs in a small area of dry rocky grassland on carbonate rocks at several closely spaced microlocalities at an altitude of about 850 m. Approximately 100 individuals (cc. 30 flowering) occur within the provisory ass. Stipo-Festucetum rubrae.

Phenology: flowering and fruiting period: May–August.

Conservation status: Since only about 30 flowering individuals have been recorded so far in the Svrljiške Mt. in eastern Serbia, in a very small dry grassland area, the new subspecies should be considered as a critically endangered taxon (CR). However, considering the larger number of potential habitats of this plant in a wider area, the actual number of individuals is likely to be higher.

### 2.4. Herbarium Specimens Examined and Field Observations

*Stipa stevanoviciorum* subsp. *stevanoviciorum*

Serbia, Mt. Zlatibor, Stublo, near Dubrava Monastery, 43.580373 N, 19.65938 E, 770 m, rocky grassland, ultramafite, 13 June 2022, E. Kabaš, S. Vukojičić, P. Lazarević, det.: E. Kabaš, sub. *S.dasyphylla* (Lindem.) Trautv. (BEOU-KEGB 71001).

Serbia, Mt. Zlatibor, Stublo, near Dubrava Monastery, 43.581646 N, 19.660864 E, 790 m, rocky grassland, serpentinite, 27 September 2021, E. Kabaš, P. Lazarević, det.: E. Kabaš, sub. *S.dasyphylla* (Lindem.) Trautv.

Serbia, Mt. Zlatibor, Stublo, near Dubrava Monastery, 43.5806821 N, 19.6596977 E, 756 m, rocky grassland, serpentinite, 27 September 2021, E. Kabaš, P. Lazarević, det.: E. Kabaš, sub. *S.dasyphylla* (Lindem.) Trautv.

*Stipa stevanoviciorum* subsp. *pseudodasyphylla*

Serbia, agrest. Niša [surroundings of Niš], 1879, S. Petrović, MW0761191, det. Smirnov sub. *S.dasyphylla* Czern.

Serbia, Niševačka N 34, *Stipa graffiana* Pleš?, 1879, S. Petrović, 2039, det. Smirnov sub. *S. dasyphylla* Czern.

## 3. Discussion

The *Stipa* species belonging to the series Dasyphyllae represent a natural, clearly defined group within the section *Stipa*, whose adaxial leaves are hairy, making it easy to distinguish them from the other groups in the section. However, some doubts have been expressed regarding determining the interspecific relations between these taxa [34]. In [34], seven taxa are even listed within the so-called *Stipa rubens* complex, emphasising the need for a thorough revision of the localities where *S. dasyphylla* has previously been reported and pointing out that the presence of *S. dasyphylla* cannot be excluded from the area of south-eastern Europe. This statement proved to be correct in the light of the findings of our study. Moreover, Martinovský [34] considered the taxa of the *S. dasyphylla* group as phylogenetically young and morphologically characterized by minor but erratic, seamless distinctive features. This also seems to be the case with our new taxa from Serbia. *S. stevanoviciorum* is probably another of these phylogenetically young taxa that have diversified within the small, isolated relic islands that present the remnants of the true steppes in the study area. On the other hand, more recent research [32] has narrowed the group down to just two species, *S. dasyphylla* being one and *S. zalesskii* the other (including *S. pontica* and *S. ucrainica* on the subspecies level). Either way, it is an uncontroversial opinion that *S. dasyphylla* has always been considered a good taxon distinguished from the related taxa by its unique characteristics. These very properties, such as hairy silvery leaves that are soft to the touch, lead us to think that populations from Serbia fall within the variety of *S. dasyphylla* [40]. However, a closer examination of the herbarium material and especially of the plants in the field, which include *S. dasyphylla* and related taxa in the wider area of Serbia, North Macedonia, Greece, Bulgaria, Romania, Hungary, Slovakia, and the Czech Republic, led us to the conclusion that the plants from Serbia are clearly different from what was considered typical for *S. dasyphylla*, as well as from other representatives of the series *Dasyphyllae*. This was later confirmed by the results of our morphometrica analyses. PCA showed a high degree of separation of Serbian populations of *S. dasyphylla*, while “typical” populations from Central Europe were associated with the remaining populations of *S. ucrainica* and *S. pontica* from North Macedonia, Greece, Bulgaria, and Romania. Moreover, DCA analysis and UPGMA cluster analysis not only confirmed the results of the PCA, but also showed significant morphological differentiation between two Serbian populations. The extent and quality of the morphological differences, together with a very pronounced disjunct distribution, provided us with sufficient arguments to split the populations from Serbia into a new species, *S. stevanoviciorum*, with two subspecies. The most significant differences that are recognisable with the naked eye and under the microscope concern the ornamentation of the leaf blade and the habit. The specimens collected in Serbia which correspond to *S. stevanoviciorum* are more robust, have a larger leaf diameter, and are equipped with dense, long, and tender hairs, which makes them soft to the touch. Nevertheless, at first glance, *S. stevanoviciorum* and its subspecies can still easily be confused with *S. dasyphylla*, to which it is the most closely related. However it differs from the latter by a number of qualitative characters: by having pubescent culms, a pubescent basal internode, a pubescent panicle basal internode, glabrous to papillose upper sheaths, mostly both flat and convolute leaves present in the tuft, as well as the length of the upper cauline leaves (125–264 mm vs. 35–160 mm), the length of the dorsal row of lemma hairs (6–10 mm vs. 3–6 m), the distance from the end of the ventral line of hairs to the top of the anthecium (0–0 vs. 0–5), the length of the hairs on the seta (5–7.5) mm vs. (3.5–6 mm), and the length ratios of different characters (Table 3).

Although the cluster analysis revealed considerable morphological divergence between the two newly proposed subspecies, we have applied the concept of subspecies here for the following reasons. First, the results of the PCA and CDA show that the overlaps on the first two principal component and discriminant axes are very pronounced, as well as that a slight separation occurs only on the third CDA axis, which describes only 6.84% of the total discrimination. In addition, most of the quantitative characters overlap, and only a few characters show a significant tendency towards separation. At the same time, these two groups of populations (i.e., the newly proposed subspecies) show a very strong ecological and geographical differentiation. The fact that, in addition to subtle morphological differences, the populations from western and eastern Serbia also differ geologically (carbonate vs. ultramafic substrate), climatically (humid temperate vs. dry subcontinental climate), and historically–biogeographically (Illyrian vs. Moesian phytoprovince) is, in our opinion, a strong argument in favour of giving them each a separate taxonomic status at the subspecies level.

Key to the taxa:

1a. Leaves always convolute, 0.5–0.9 mm in diameter, abaxial surface aculeolate and with sparse, subappressed hairs.........................................….……………….………...………2

1b. Leaves flat or convolute, when convolute diameter c. 1 mm, with dense, long hairs, soft to the touch..................................................................................................……….…3

2a. Ventral line of hairs on lemma ending 2–6 mm below the insertion of the awn, sheaths mostly scabrid, leaf diameter c. 0.5 mm.....................................................*S. ucrainica*

2b. Ventral lines of hair on lemma ± reaching the insertion of the awn, sheaths glabrous, leaf diameter 0.5–0.9 mm..................………………….……….……................*S. pontica*

3a. Anthecium 19–24 mm, length of the dorsal row of lemma hairs 6–10 mm, length of upper cauline leaves 35–160 mm.........................................................................*S. dasyphylla*

3b. Anthecium 22–25 mm, length of the dorsal row of lemma hairs 3–6 mm, length of upper cauline leaves 125–264 mm................................................................................................4

4a. Plant height 40–57 cm, number of flowers in panicle 7–10, length of the culm leaf ligule 2.5–4 mm, ligule tip ciliolate, column brown......................................*S. stevanoviciorum* subsp. *stevanoviciorum*

4b. Plant height 28–42 cm, number of flowers in panicle 4–8, length of the uppermost culm leaf ligule 6–8 mm, ligule tip ciliate, column yellow........................*S. stevanoviciorum* subsp. *pseudodasyphylla*

## 4. Materials and Methods

### Plant Material and Morphometric Analyses

Morphometric analyses were performed on the material collected in the field as well as on herbarium specimens deposited in BEOU, BEO, and MW (herbarium abbreviations follow [40]). In addition, various virtual herbaria and GBIFs were reviewed (see Appendix A for links).

The morphometric study included 160 individuals from 17 populations belonging to three “dasyphyllous” Stipa species (*S. pontica*, *S.ucrainica*, and *S.dasyphylla*) collected in the central and eastern Balkan Peninsula and Central Europe (Table 4). In this study, we included taxa previously classified in the *S. dasyphylla* group, as outlined in *Flora Europaea* [7], which are characterised by a densely hairy adaxial leaf surface and an abaxial surface with conical papillae that are often accompanied by 0.5–1 mm-long hairs, and which occur in Europe.

From each population, from 6 to 15 plant samples were used for morphometric analyses. The voucher specimens were deposited in the Herbarium of the Institute of Botany and Botanical Garden, of the Faculty of Biology, University of Belgrade (BEOU). The morphological characteristics were observed and measured using a stereo microscope Leica MZ 7_5_ and photographed using Leica DFC 295 camera with Leica Application Suite (LAS) Version 4.12.0.

A total of 37 morphological characters were measured (24 quantitative and 13 qualitative), which are listed in Table 5. Descriptive statistics (minimum and maximum values, mean, standard deviation, and coefficient of variation) were calculated for each quantitative morphometric characteristic measured. Prior to all multivariate analyses, the morphometric data were transformed using Box-Cox transformation in PAST 4.17 [41]. In order to eliminate a single characteristic from highly correlated pairs, pairwise Spearman correlation analyses were performed, and four characters with a Spearman correlation coefficient greater than 0.80 were excluded from the multivariate analysis. Different multivariate approaches were used to compare all characters and groups assessed, including principal component analysis (PCA), canonical discriminant analysis (CDA), and cluster analysis using Statistica v.8.0 (StatSoft and Inc 2007). Principal component analysis (PCA) was performed to reveal the overall morphological variation and relationships between individuals in all populations. Canonical discriminant analysis (CDA) was used to test the hypothesis of morphological segregation of six groups of individuals identified a priori as potential OTUs based on their taxonomic and biogeographic affiliations. *S. ucrainica* is divided into two groups: *S. ucrainica*-BU-RU (Bulgaria and Romania) from the Danube phytoprovince and *S. ucrainica*-MA (North Macedonia) from the Macedonian phytoprovince. *S. dasyphylla* is divided into three groups: *S. dasyphylla*-C-EU (Central Europe) from the Pannonian phytoprovince, *S. dasyphylla*-SR-W (Serbia, western) from the Illyrian phytoprovince, and *S. dasyphylla*-SR-E (Serbia, eastern) from the Balkan (Moesian) phytoprovince. Finally, *S. pontica* from the Thracian phytoprovince is defined as a separate group (Table 4). The biogeographical affiliation follows the phytogeographical map of Europe [42], in which each phytoprovince represents a unit defined by historical, ecological, and geographical barriers, each with different floristic and ecosystem characteristics (Table 4). Finally, the UPGMA (unweighted pair group method with arithmetic mean) cluster analysis based on Mahalanobis distances between all analysed groups was computed, the classification function was used to determine the percentage of correctly classified individuals in each group, and discriminant function analysis (DFA) was used to estimate the contribution of individual characters to the overall differentiation. The threatened status of *S. stevanoviciorum* in Serbia was assessed using the IUCN criteria [43].

## Figures and Tables

**Table 1:** The classification function based on morphological characters for six a priori-identified groups of “dasyphyllous” *Stipa* from the Balkan Peninsula.

	Percent Correct	1	2	3	4	5	6
1. *S.ucrainica*-BU-RU	93.88	46	3	0	0	0	0
2. *S.ucrainica*-MA	72.73	3	8	0	0	0	0
3. *S.pontica*	100.00	0	0	20	0	0	0
4. *S.dasyphylla*-C_EU	100.00	0	0	0	60	0	0
5. *S.dasyphylla*-SR-W	100.00	0	0	0	0	10	0
6. *S.dasyphylla*-SR-E	100.00	0	0	0	0	0	10
Total	96.25	49	11	20	60	10	10

**Table 2:** Factor loadings of the first three principal component axes and discriminant function analysis (DFA) based on morphological characters for six a priori-identified groups of “dasyphyllous” Stipa from the Balkan Peninsula.

	Factor Loadings			Discriminant Function Analysis			
	PCA1	PCA2	PCA3	Wilks’ Lambda	Partial Lambda	F-Remove	P-Level
PH_Plant height (cm)	−0.714	0.318	0.197	0.002	0.829	5.571	0.0001
LP_length of panicle	−0.767	0.295	0.250	0.001	0.968	0.898	0.4847
LG_Length of the lower glume (mm)	−0.575	−0.244	0.100	0.002	0.798	6.843	0.0000
NF_number of flowers in panicle	−0.734	0.293	0.130	0.001	0.859	4.447	0.0009
LCL_length of upper cauline leaves (mm)	−0.691	−0.235	0.245	0.002	0.618	16.671	0.0000
LUL_Ligule uppermost leaf length (mm)	−0.311	−0.148	−0.058	0.002	0.720	10.478	0.0000
LL_Basal ligule length (mm)	−0.429	0.204	−0.361	0.001	0.961	1.098	0.3643
LV_length of vegetative shoots (mm)	−0.811	0.338	0.066	0.001	0.866	4.184	0.0014
BASD_Basal leaf-blade diameter (mm)	−0.707	−0.043	−0.389	0.002	0.662	13.785	0.0000
AL_length of anthecium (mm)	−0.881	−0.246	0.009	0.002	0.812	6.240	0.0000
DL_Dorsal row length (mm)	0.102	−0.806	0.282	0.002	0.645	14.837	0.0000
DDL_distance from the end of dorsal line of hairs to the top of anthecium (mm)	−0.741	0.284	−0.210	0.002	0.832	5.467	0.0001
DVL_distance from the end of ventral line of hairs to the top of anthecium (mm)	0.800	0.101	0.345	0.002	0.697	11.729	0.0000
WA_width of anthecium (mm)	−0.546	−0.437	−0.403	0.001	0.953	1.340	0.2511
AWND_Awn diameter (mm)	0.073	0.001	−0.538	0.001	0.932	1.985	0.0847
PERL_Peripheral ring length (mm)	−0.183	−0.582	0.220	0.001	0.873	3.941	0.0023
PERW_peripheral ring width (mm)	−0.246	−0.166	−0.271	0.001	0.962	1.056	0.3878
CAL_Callus length (mm)	−0.592	−0.348	0.200	0.001	0.953	1.343	0.2501
AWN_Awn length (cm)	−0.439	0.382	0.570	0.002	0.753	8.878	0.0000
SHL_Length of hairs on seta (mm)	−0.362	−0.610	0.086	0.002	0.790	7.182	0.0000

**Table 3:** Main differences between *Stipa dasyphylla* and subspecies of *S. stevanoviciorum*. The most significant diagnostic characters are given in boldface (* diagnostic between *S. dasyphylla* and *S. stevanoviciorum*, ** diagnostic between subspecies of *S. stevanoviciorum*). Quantitative continuous characters and ratios are reported as mean ± standard deviation, with the minimum and the maximum values in brackets.

	*S. Dasyphylla*	*S. Stevanoviciorum* *Stevanoviciorum*	*S. Stevanoviciorum* *Pseudodasyphylla*
**PH_Plant height (mm)	(245–)368–580(–703)	**(353–)407** **–564(–595)**	**(223–)280** **–424(–460)**
*CO_Culm ornamentation	scabrous, rarely pubescent	pubescent	pubescent
LP_length of panicle (mm)	(58–)89–141(–180)	(85–)92–169(–185)	(70–)66–118(–155)
LG_Length of the lower glume (mm)	(39–)58–80(–92)	(61–)64–76(–78)	(73–)75–101(–120)
UG_Length of the upper glume (mm)	(30–)48–70(–77)	(54–)54–69(–75)	(70–)67–91(–110)
**NF_number of flowers in panicle	(4–)7–11(–14)	**(7–)7**–**11(–12)**	**(4–)4**–**9(–12)**
PA_Panicles	exerted to partially enclosed	exerted	exerted to partially enclosed
*PBI_Panicle basal internode surface	scabrous, rarely pubescent	pubescent	pubescent
*/**LCL_length of upper cauline leaves (mm)	(35–)49–103(–160)	**(159–)176**–**237(–264)**	**(125–)135**–**175(–190)**
**LUL_Ligule uppermost culm leaf length (mm)	(2–)3–5(–8)	**(2.5–)2.7**–**3.8(–4)**	**(6–)6.6**–**9.5(–11)**
*/**UPO_Upper sheaths ornamentation	Papillose to scabrous with stiff hairs, rarely glabrous or pubescent	**papillose**	**glabrous to papillose**
**LL_Basal ligule length (mm)	(1–)2–4(–6)	**(1–)1**–**2(–2)**	**(2–)2**–**4(–4)**
*BLM_Basal leaf ligule margin	ciliate to ciliolate, rarely glabrous	glabrous	glabrous
**BLT_ligule tip	ciliate to ciliolate	**ciliolate**	**ciliate**
LV_length of vegetative shoots (mm)	(393–)499–705(–819)	(417–)427–584(–698)	(394–)412–604(–699)
BASD_Basal leaf–blade diameter (mm)	(1–)1–1(–1)	(1–)1–1(–1)	(1–)1–1(–1)
**LBT_type of basal leaves in sward	in some individuals only flat or only convolute leaves present	**both flat and convolute leaves always present**	**in some individuals only flat or only convolute leaves present**
ABIND_Abaxial surface of basal blade–leaf	sparsely to densely soft hairs	sparsely to densely soft hairs	sparsely to densely soft hairs
AL_length of anthecium (mm)	(18–)19.4–21.8(–23)	(21–)21.9–24.6(–25.5)	(21.5–)22.3–24(–24)
ML_Ventral row length (mm)	(11–)14.6–17.1(–19)	(17–)17.6–20.2(–21)	(16–)17.1–18.9(–19)
*DL_Dorsal row length (mm)	(3–)3.2–4.8(–6)	(6–)6.8–9.2(–10)	(6–)6.7–9.1(–10)
*DVL_distance from the end of ventral line of hairs to the top of anthecium (mm)	(0–)–0.7–1.2(–5)	(0–)0–0(–0)	(0–)0–0(–0)
DDL_distance from the end of dorsal line of hairs to the top of anthecium (mm)	(10–)10.8–13.3(–15)	(9–)9.9–11.9(–13)	(8–)8.9–11.3(–12)
WA_width of anthecium (mm)	(1–)1–1.5(–1.8)	(1–)1.3–1.6(–1.5)	(1–)1.3–1.8(–2)
AWND_Awn diameter (mm)	(0.5–)0.5–0.6(–0.8)	(0.5–)0.5–0.5(–0.5)	(0.5–)0.5–0.5(–0.5)
PERL_Peripheral ring length (mm)	(0.5–)0.9–1.1(–1.2)	(1–)1–1.4(–1.5)	(1–)1–1.2(–1.3)
PERW_peripheral ring width (mm)	(0.4–)0.5–0.5(–0.5)	(0.5–)0.5–0.5(–0.5)	(0.5–)0.5–0.5(–0.5)
CAL_Callus length (mm)	(2–)3.1–4.7(–6.5)	(4–)4.3–4.9(–5)	(5–)4.9–6.4(–7.5)
AWN_Awn length (mm)	(250–)305.2–395.7(–454)	(273–)294.4–348.6(–357)	(287–)296.8–378.4(–435)
COL_Column length (mm)	(65–)73.9–93.5(–105)	(81–)84.2–97.2(–102)	(93–)93.2–103.4(–108)
ACS_awns column surface	glabrous	glabrous	glabrous
**CC_Colour of column	brown to yellow	**brown**	**yellow**
SET_Seta length (mm)	(174–)229–306(–354)	(186–)207–254(–268)	(193–)203–276(–327)
*SHL_Length of hairs on seta (mm)	(3.5–)4.2–5.4(–6)	**(5.5–)5.3**–**7(–7.5)**	**(5–)5.3**–**6.8(–7.5)**
PERL/PERW_Ratio of peripheral ring length and peripheral ring width	(1.3–)1.8–2.2(–2.5)	(2–)2.1–2.8(–3)	(2–)2–2.5(–2.5)
*DL/AL_Ratio dorsal row length and anthecium length	(0.1–)0.2–0.2(–0.3)	**(0.3–)0.3**–**0.4(–0.4)**	**(0.3–)0.3**–**0.4(–0.4)**
*SET/COL_Ratio length of seta to the length of column	(2.3–)2.8–3.6(–4.5)	**(2.1–)2.3**–**2.8(–3)**	**(2.1–)2.2**–**2.7(–3)**

**Table 4:** Studied populations of *Stipa ucrainica*, *S. dasyphylla*, and *S. pontica*.

No.	Country and Locality	Geographic Code	Coordinates	Elev., M	Collector(s)	N_herb_
	*Stipa ucrainica*					
P01	Bulgaria, Tylenovo	BU-RU	43.487211 N, 28.528549 E	43	Kabaš, E., Vukojičić, S., Lazarević, P.	67581
P02	Bulgaria, Pomorie	BU-RU	42.637393 N, 27.556744 E	112.5	Kabaš, E., Vukojičić, S., Lazarević, P.	67594
P03	Romania, Babadag	BU-RU	44.817343 N, 28.699467 E	128.6	Kabaš, E., Vukojičić, S., Lazarević, P.	67553
P04	Romania, Viteazu Mihai	BU-RU	44.58028 N, 28.637563 E	110.6	Kabaš, E., Vukojičić, S., Lazarević, P.	67567
P05	Romania, Gura Dobrogei	BU-RU	44.489989 N, 28.57492 E	146.6	Kabaš, E., Vukojičić, S., Lazarević, P.	67568
P06	N Macedonia, Veles-Katlanovo	MA	41.817471 N, 21.694689 E	457	Kabaš, E., Arsovska, R.	41824
P07	N Macedonia, Vetersko	MA	41.814399 N, 21.695896 E	431.3	Kabaš, E., Đurović, S., Ćušterevska, R.	67622
	*Stipa dasyphylla*					
P08	Hungary, Matra	C-EU	47.778767 N, 19.709707 E	310	Kabaš, E., Lazarević, P.	71056
P09	Hungary, Bukk Nacionalni park	C-EU	47.915343 N, 20.440163 E	320	Kabaš, E., Lazarević, P.	71055
P10	Romania, Gorge Turda	C-EU	46.5635843 N, 23.689981 E	454	Kabaš, E., Lazarević, P.	71062
P11	Slovakia, Obyce	C-EU	48.450428 N, 18.478622 E	435	Kabaš, E., Lazarević, P.	71073
P12	Slovakia, Plaštovce	C-EU	48.171553N, 18.969552E	417	Kabaš, E., Lazarević, P.	71075
P13	Czech, Mohelno	C-EU	49.109387 N, 16.183609 E	100	Kabaš, E., Lazarević, P.	71065
P14	Serbia, Svrljiške mountain	SR-E	43.30276 N, 22.30041 E	862	Kabaš, E., Stanković, V., Đorđević, V.	71076
P15	Serbia, Zlatibor	SR-W	43.580373 N, 19.65938 E	776	Kabaš, E., Vukojičić, S., Lazarević, P.	71001
	*Stipa pontica*					
P16	Bulgaria, Orahovo	-	41.923037 N, 26.13183 E	229	Kabaš, E., Lazarević, P., Stanković, V.	72401
P17	Greece, Kiprinos	-	41.588074 N, 26.299067 E	58	Kabaš, E., Lazarević, P., Stanković, V.	72402

**Table 5:** Morphological characters measured in this study. Characters with asterisk (*) are included in the multivariate analysis; highly correlated characters excluded from the multivariate analysis are marked with two asterisks (**).

No.	Abbreviation	Character Description
1	ABIND	Abaxial surface of basal blade-leaf: glabrous (0), minutely scabrous (1), distinctly scabrous (2), sparsely stiff hairs (3), + “sparsely to densely soft hairs (4)”
2	ACS	awns column surface: hairy (0), glabrous (1), tuberculate (2).
3	AL	* Length of anthecium (mm); measured from the base of the lemma to the beginning of the column
4	AWN	* Awn length (cm); measured in the widest part
5	AWND	* Awn diameter (mm); the width of the column was measured, a few millimeters above the lemma
6	BASD	* Basal leaf-blade diameter (mm); measured in the lower half of the basal leaf
7	BLM	Basal leaf ligule margin: glabrous (0), ciliate (1), ciliolate (2)
8	BLT	Ligule tip: glabrous (0), ciliate (1), ciliolate (2)
9	CAL	* Callus length (mm); measured from the base of the callus to the beginning of the lemma
10	CC	Color of column (1—green, 2—light-red, 3—red-purple, 4—brown, 5—yellow)
11	CO	Culm ornamentation: glabrous (0), scabrous (1), pubescent (2)
12	COL	** Column length (cm); measured from the top of the lemma to the beginning of the seta
13	CS	Callus shape: slightly curved (0), straight (1)
14	DDL	* Distance from the end of dorsal line of hairs to the top of anthecium (mm), measured from the top of the dorsal hairline to the top of the lemma
15	DL	* Dorsal row length (mm); measured from the beginning of the lemma to the end of the dorsal hairline
16	DSDF	Dorsal and subdorsal free: no (0), yes (1)
17	DVL	* Distance from the end of ventral line of hairs to the top of anthecium (mm), measured from the top of the ventral hairline to the top of the lemma
18	LBA	* Leaf-blades apex: glabrous or scabrous (0), finished in a tassel of hairs (1), long acuminate (2)
19	LBT	Type of basal leaves in sward: “only convolute leaves present (1)“, “only flat leaves present (2)”, “both flat and convolute leaves present (3)”
20	LCL	* Length of upper cauline leaves (mm); measured from the base of the upper ligule to the top of the leaf
21	LG	* Length of the lower glume (mm); measured from the base of the lower glume to its peak
22	LL	* Basal ligule length (mm); measured from the base to the tip of the first basal ligule
23	LP	* Length of panicle; measured from the base of the inflorescence to the base of the last flower
24	LUL	* Ligule uppermost leaf length (mm); measured from the upper ligule base to its top
25	LV	* Length of vegetative shoots (mm); measured from the base to the tip of the longest leaf
26	ML	** Ventral row length (mm); measured from the beginning of the lemma to the end of the ventral hairline
27	NF	* Number of flowers in panicle
28	PA	Panicles: exserted (0), partially enclosed (1), enclosed (2)
29	PBI	Panicle basal internode surface: glabrous (0), scabrous (1), pubescent (2)
30	PERL	* Peripheral ring length (mm)
31	PERW	* Peripheral ring width (mm)
32	PH	* Plant height (cm); measured from the the roots to the base of the inflorescence
33	SET	** Seta length (cm); measured from the end of the column to the end of the seta
34	SHL	* Length of hairs on seta (mm); measured in the middle of the seta
35	UG	** Length of the upper glume (mm); measured from the base of the upper glume to its peak
36	UPO	Upper sheaths ornamentation: glabrous (0), papillose (1), scabrous (2), scabrous with stiff hairs (3), + “pubescent (4)”
37	WA	* Width of anthecium (mm); measured at the lower half of the anthecium, where it is widest

## Data Availability

The original contributions presented in this study are included in the article. Further inquiries can be directed to the corresponding author.

## Abbreviations

The following abbreviations are used in this manuscript:

BEOU	University of Belgrade
BEO	Natural History Museum
MW	Moscow State University
GBIF	Global Biodiversity Information Facility
IUCN	International Union for Conservation of Nature

## Appendix A

**Table A1:** The links to virtual herbaria consulted for the purposes of this study.

The Links to Virtual Herbaria
http://apps.kew.org/herbcat/gotoHomePage.do (accessed on 17 March 2025).
http://herbarium.agr.hr/search.html (accessed on 17 March 2025).
http://herbarium.emg.umu.se/index.html (accessed on 17 March 2025).
http://ww2.bgbm.org/herbarium/ (accessed on 17 March 2025).
https://bioportal.naturalis.nl/ (accessed on 17 March 2025).
https://coicatalogue.uc.pt/ (accessed on 17 March 2025).
https://data.kew.org/?lang=en-US (accessed on 17 March 2025).
https://data.nhm.ac.uk/dataset/collection-specimens/resource/05ff2255-c38a-40c9-b657-4ccb55ab2feb?view_id=6ba121d1-da26-4ee1-81fa-7da11e68f68e&filters=collectionCode%3ABOT (accessed on 17 March 2025).
https://data.rbge.org.uk/search/herbarium/ (accessed on 17 March 2025).
https://explore.recolnat.org/search/botanique/type=index (accessed on 17 March 2025).
https://gallery.hungaricana.hu/hu/search/results/?list=eyJxdWVyeSI6ICJHWVVKVEVNRU5ZPShIZXJiYXJpdW0pIn0 (accessed on 17 March 2025).
https://herbarium.nrm.se/search/species/?query=Hieracium (accessed on 17 March 2025).
https://herbier2014.univ-lyon1.fr/search (accessed on 17 March 2025).
https://hirc.botanic.hr/fcd/search.aspx (accessed on 17 March 2025).
https://hrcak.srce.hr/file/45425 (accessed on 17 March 2025).
https://kiki.huh.harvard.edu/databases/specimen_index.html (accessed on 17 March 2025).
https://pancic.bio.bg.ac.rs/Yu/Herbar/Upit.html (accessed on 17 March 2025).
https://plant.depo.msu.ru/?d=P (accessed on 17 March 2025).
https://plants.jstor.org/search?asf= (accessed on 17 March 2025).
https://science.mnhn.fr/institution/mnhn/search (accessed on 17 March 2025).
https://scientific-collections.gbif.org/specimen/search?view=GALLERY (accessed on 17 March 2025).
https://ww2.bgbm.org/Herbarium/ (accessed on 17 March 2025).
https://www.europeana.eu/en (accessed on 17 March 2025).
https://www.europeana.eu/et/search?query=Cerastium%20decalvans (accessed on 17 March 2025).
https://www.gbif.de/de/search/node (accessed on 17 March 2025).
https://www.gbif.org/species/search (accessed on 17 March 2025).
https://www.herbarien.uzh.ch/en/belegsuche.html (accessed on 17 March 2025).
https://www.jacq.org/#database (accessed on 17 March 2025).
https://www.omnia.ie/index.php (accessed on 17 March 2025).
https://www.ville-ge.ch/musinfo/bd/cjb/chg/advanced.php (accessed on 17 March 2025).
https://www.botanicalcollections.be/#/en/search/specimen (accessed on 17 March 2025).
https://www.ville-ge.ch/musinfo/bd/cjb/chg/index.php?lang=en (accessed on 17 March 2025).
https://data.nhm.ac.uk/search (accessed on 17 March 2025).
https://data.kew.org/?lang=en-US (accessed on 17 March 2025).
https://gwdu64.gwdg.de/pls/herbar/typen$.startup (accessed on 17 March 2025).
https://www.nmnhs.com/e-natura/types-bulgaria/type_list_institution_bg-SOA-p-1.html (accessed on 17 March 2025).
https://plant.depo.msu.ru/open/public/en/collections (accessed on 17 March 2025).
https://www.nmnhs.com/recent-plants-collection-en.html (accessed on 17 March 2025).
https://herbarium.nrm.se/search/specimens/?name=potamogeton%20nodosus (accessed on 17 March 2025).
